# The role of macrophage subsets in and around the heart in modulating cardiac homeostasis and pathophysiology

**DOI:** 10.3389/fimmu.2023.1111819

**Published:** 2023-02-28

**Authors:** Carmina Albertine Isidoro, Justin F. Deniset

**Affiliations:** ^1^ Department of Physiology and Pharmacology, University of Calgary, Calgary, AB, Canada; ^2^ Libin Cardiovascular Institute, Cumming School of Medicine, Calgary, AB, Canada; ^3^ Department of Cardiac Sciences, University of Calgary, Calgary, AB, Canada

**Keywords:** macrophages, tissue-resident, cardiac, pericardial cavity, remodeling

## Abstract

Cardiac and pericardial macrophages contribute to both homeostatic and pathophysiological processes. Recent advances have identified a vast repertoire of these macrophage populations in and around the heart - broadly categorized into a CCR2^+^/CCR2^-^ dichotomy. While these unique populations can be further distinguished by origin, localization, and other cell surface markers, further exploration into the role of cardiac and pericardial macrophage subpopulations in disease contributes an additional layer of complexity. As such, novel transgenic models and exogenous targeting techniques have been employed to evaluate these macrophages. In this review, we highlight known cardiac and pericardial macrophage populations, their functions, and the experimental tools used to bolster our knowledge of these cells in the cardiac context.

## Introduction

The heart plays an integral physiological role to distribute oxygen and nutrient-rich blood to the rest of the body. This requires a constant coordinated action of cardiac muscle cells, or cardiomyocytes, that will coordinate your heart to beat 4 billion times over your lifespan. In the heart, cardiomyocytes are noted as the work horse of this system. Growing research has identified heterogeneous supporting cells, such as stromal and immune cells, that populate the tissue under normal conditions. The pericardial cavity, which plays an important mechanical and lubricating role for the heart, is equally populated by a reservoir of immune cells. Tissue-resident macrophages, like in many organs, have been identified as a key immune component of this support system in the heart with the ability to mediate development, facilitate cellular functions of the cardiomyocytes, and serve as sentinel cells under inflammatory conditions. Changes to the local microenvironment following direct injury to the heart or peripheral conditions can engender different remodeling processes that can contribute to the progression to heart failure when the heart can no longer meet the demands of the rest of the body. This review will explore how cardiac and pericardial macrophage subsets contribute to both homeostatic and remodeling settings in the heart, in addition to the tools that we employ to evaluate these functions in mice.

## Cardiac and pericardial macrophage phenotypes and origins

**Figure 1:**
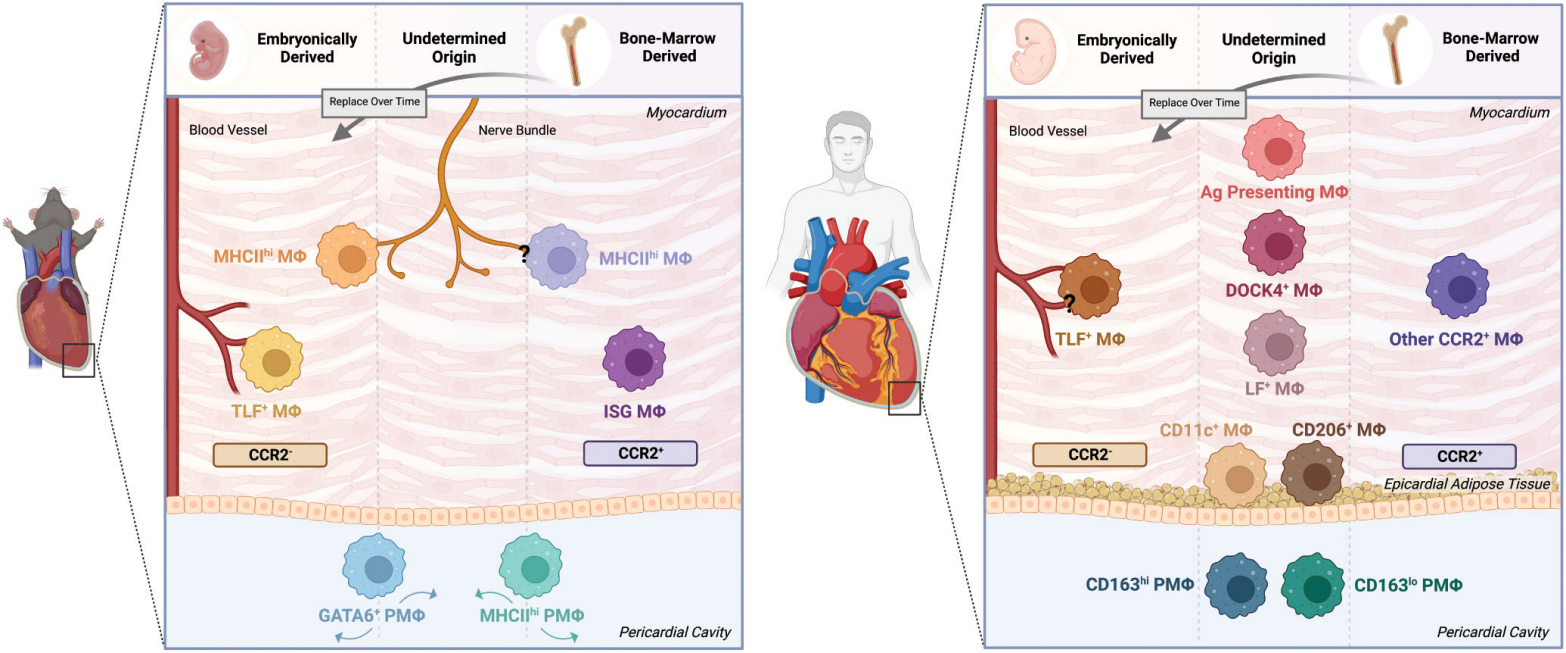
Cardiac and pericardial macrophage populations in mice and humans. The macrophage lineage has been established to give rise to heterogeneous populations. As such, this heterogeneity applies to both cardiac macrophage (MΦ) and pericardial macrophage (PMΦ) populations in the mouse and human contexts. In mice, lineage tracing techniques have identified embryonically-derived and bone marrow-derived cardiac MΦ populations. Embryonically-derived cardiac MΦ include CCR2^+^MHC^hi^ and CCR2^+^TimD4^+^Lyve1^+^FolR2^+^(TLF^+^) populations localized around nerve bundles and blood vessels, respectively. Bone marrow-derived populations include the nerve bundle localized CCR2^-^MHC^lo^ MΦ and the CCR2^-^ISG MΦ. The pericardial cavity in mice is also a niche of unique macrophage populations, specifically GATA6^+^ and MHC^hi^ PMΦ. However, the origin of these cells have yet to be confirmed. In humans, identifying the phenotype, origin and localization of macrophages becomes more difficult. Similar to mice, humans have comparable embryonically-derived perivascular CCR2^-^TLF^+^ MΦ and bone marrow-derived CCR2^+^ MΦ. Yet, while other transcriptionally and functionally unique populations have been identified, their origins and localizations remain uncertain. These populations include but not limited to DOCK4^+^, Lyve1^+^FolR2^+^(LF+), and Antigen (Ag) Presenting MΦ. Likewise, PMΦ of undetermined origin also exist, dichotomized into CD163^hi^ and CD163^lo^ populations. The epicardial adipose tissue in humans is also home to MΦ subsets broadly characterized as CD11c^+^ and CD206^+^ populations. Created with BioRender.com.

Interest in macrophages in the heart once centered around their involvement in the inflammatory response following an injury. However, the recognition of tissue-resident macrophages with homeostatic functions in many organs has sparked exploration into the heart and surrounding pericardium in both mice and humans. Numerous single cell RNA sequencing studies (**Supplementary Table 1**) combined with advances in transgenic mouse tools have helped to establish the macrophage populations, their localization, and their origins (**Figure 1**).

### Heart

Cardiac-resident macrophages (CRM) in mice were first collectively characterized by their expression of the chemokine receptor Cx_3_CR1, insulin growth factor-1 (IGF1), interleukin (IL)-10, and a high expression of Lyve1, as well as their localization alongside blood vessels and cardiomyocytes (1). This initial view was then expanded to include multiple populations with different origins and locations that can be distinguished in large part on the expression and/or absence of MHCII and CCR2 markers. CCR2^-^ CRM are the predominant population of macrophages in the heart and can be further divided in to MHCII^lo^ and MHCII^hi^ subsets. Dick et al. have further characterized MHCII^lo^ CCR2^-^ CRM that includes a trio of markers (TimD4^+^, Lyve1^+^ and FolR2^+^) and as such have termed them TLF^+^ macrophages (2). Lineage tracing and parabiosis approaches demonstrate that both TLF^+^ and MHCII^hi^ CRM derive from yolk sac and fetal liver sources and self-maintain locally with minimal contribution from monocytes during homeostatic conditions (2–6). During embryonic development, Lyve1^+^ embryonic macrophages are first seen to localize to the epicardial surface and are proposed to give rise to CRM (7). It is unclear whether these epicardial cells give rise to both CCR2^-^ CRM lineages or simply the TLF^+^ population; however, in adult mice, these embryonically-derived populations have minimal input to each other and do not appear to represent different stages along a maturation continuum (5). These distinct lineages also reside in different locations in the heart, with TLF^+^ CRM adopting a perivascular location and MHCII^hi^ CRM enriched around nerve bundles (5). The cells and mediators from these local milieus that maintain these observed phenotypes are not well understood. Interactions with other immune cells may impact this process, as B cell deficiency contributes to lower MHCII^hi^ CCR2^-^ CRM numbers in the heart (8). Conversely, the absence of the cell surface receptor MERTK involved in apoptotic body/cell uptake and CRM homeostatic functions (discussed below) contributes to an increase proportion of CCR2^-^ MHCII^hi^ CRM (9). CCR2^+^ CRM are found in very low abundance during development in mice but increase over time and are maintained by circulating monocytes (3, 4, 10). It should be noted that these CCR2^+^ CRM also express high levels of MHCII (3), and as such, have likely been bundled with CCR2^-^MHCII^hi^ CRM in many earlier studies where CCR2 was not incorporated in the analysis. To this point, it is currently unknown whether MHCII^hi^ CRM that are localized to nerve bundles include both CCR2^-^ and CCR2^+^ populations. Future imaging studies that integrate the same markers used to distinguish these populations by flow cytometry or scRNAseq are needed to better define their localization. Heterogeneity may also exist amongst the CCR2 CRM. Recently, a fourth macrophage population that carries an interferon responsive gene signature has been identified by single cell RNAseq (scRNAseq) in the steady state condition (2). Genes central to this CRM signature were previously included in the larger CCR2^+^ CRM compartment evaluated by bulk RNAseq of sorted cells (4).

### Pericardial cavity

Beyond the heart tissue, the mouse pericardial cavity surrounding the heart is populated by two main macrophage populations: Gata6^+^ pericardial and MHCII^+^ pericardial macrophages (11). These populations are reminiscent of similar populations in both the pleural and peritoneal cavities in mice (11–15). In fact, bulk RNAseq of sorted Gata6^+^ macrophages from the 3 cavities (pericardial, pleural, peritoneal) demonstrates a conserved transcriptome profile across these spaces. In contrast, Gata6^+^ pericardial macrophages differ greatly from overall CRM (largely CCR2^-^) (11). Due to these similarities with other cavities, potential cues that drive these pericardial populations can be gleaned from previous work done primarily in the peritoneal space. Okabe et al. established retinoic acid along with other omental derived signals as being key drivers of the cavity macrophage signature, including Gata6 expression (15). WT1^+^ expressing mesothelial and stromal cells, found in all organs surrounded by a cavity, are key regulators of the local retinoic acid metabolism that support Gata6 expression and Gata6^+^ macrophage maintenance (12). Lineage tracing experiments have shown that peritoneal Gata6^+^ macrophages are originally from an embryonic origin but are replaced by bone marrow derived sources over time (16, 17). In contrast, MHCII^+^ cavity macrophages in the pleural and peritoneal spaces appear to be dependent on monocyte-derived recruitment. CCR2-deficient mice have perturbed MHCII^+^ cavity macrophage populations and their differentiation in the peritoneal and pleural cavities is IRF4 dependent (14). Further validation of the macrophage origin and maintenance mechanisms is required in the pericardial space.

### Human populations

For translational purposes, studies have also explored whether similar populations exist in the human context. In the heart, samples evaluated thus far have been obtained from either relatively healthy (transplant heart donor) or heart failure patients; thus, likely reflect the ends of the disease continuum. Nonetheless, comparable TLF^+^ CRM, CCR2^+^ CRM, and antigen presenting (perhaps MHCII^hi^ equivalent) macrophages have been found at different stages in life and disease states (3, 18–21). Further populations, including DOCK4^+^ macrophages and described monocyte-derived macrophages that are also Lyve1^+^ and Folr2^+^, have also been identified (20). In the absence of genetic tools, Bajpai and al. cleverly adopted a naturally occurring lineage tracing system by collecting sex mix-matched transplanted heart tissue to evaluate the integration of host monocyte-derived cardiac macrophages relative to host-derived CRM (19). Their study found a low percentage of recipient derived CCR2^-^ CRM and a higher percentage of CCR2^+^ CRM, supporting similar maintenance programs uncovered in greater detail in the mouse system. In the pericardial cavity, samples taken from patients undergoing cardiac surgery show at the presence of Gata6 expressing macrophages that include both CD163^hi^ and CD163^lo^ expressing populations (22). In similar cardiac surgery patients, macrophages in the epicardial adipose tissue (EAT) have also been noted. These populations are CD68^+^ and are predominantly characterized by pro-inflammatory CD11c^+^ and reparative/anti-inflammatory CD206^+^ populations (23). Higher ratios of CD11c^+^/CD206^+^ EAT macrophages are associated with coronary artery disease development and metabolic disorders (23–26). EAT is virtually absent at steady-state conditions in mice, however, the presence of these populations has been noted in an experimental obesity mouse model (26). The growing knowledge of these different macrophage subsets, origins and relevance to the human system provide important context and a starting point to explore their functions.

## Tools to evaluate cardiac and pericardial macrophage populations

The phenotypic and spatial locations for these CRM and pericardial macrophages suggest that these populations likely play distinct roles under both healthy and disease states. Various exogenous and genetic approaches to broadly and specifically target these macrophage populations have been developed to evaluate these functions in mice (**Table 1**).

**Table 1:** Exogenous and genetic tools to target cardiac and pericardial macrophages.

Approach	Target MΦ population	Type of Manipulation	Potential Off-Target Populations	References
Exogenous Targeting				
Clodronate liposomes	All Cardiac MΦ, Gata6^+^ and MHCII^+^ Pericardial MΦ	Depletion	Monocytes	(21–24)
CD115 antagonist or antibody	CCR2^-^ Cardiac MΦ (TLF^+^, MHCII^hi^)	Depletion	Monocytes, Pericardial MΦ and other tissue MΦ	(25, 26)
CCR2 antagonist or antibody	Recruited Cardiac MΦ	Block Recruitment	CCR2^+^ Cardiac MΦ, MHCII^+^ Pericardial MΦ, CCR2^+^ leukocytes	(27)
CCR2 miRNA	Recruited Cardiac MΦ	Block Recruitment	CCR2^+^ Cardiac MΦ, MHCII^+^ Pericardial MΦ, CCR2^+^ leukocytes	(28, 29)
Adhesion molecule cocktail	Recruited Cardiac MΦ	Block Recruitment	Other leukocytes	(30)
Transgenic Models				
CCR2^-/-^	Recruited Cardiac MΦ	Block Recruitment	CCR2^+^ Cardiac MΦ, MHCII^+^ Pericardial MΦ, CCR2^+^ leukocytes	(22, 25, 26, 31–33)
CSF1^op/op^	All Cardiac MΦ	Depletion	Gata6^+^ and MHCII^+^ Pericardial MΦ, other tissue MΦ	(31, 32, 34)
CD169-DTR	CCR2^-^ Cardiac MΦ (TLF^+^, MHCII^hi^)	Depletion	Other tissue MΦ	(4, 35)
CCR2-DTR	CCR2^+^ Cardiac MΦ	Depletion	Ly6C^hi^ monocytes, CCR2^+^ leukocytes	(4)
CD11b-DTR	All Cardiac MΦ	Depletion	Other myeloid cells	(34)
Cx_3_CR1-creERT	CCR2^-^ Cardiac MΦ (TLF^+^, MHCII^hi^)	Depletion, Conditional KO	Other tissue MΦ	(2, 3, 17, 26, 31–36)
Lyve1^cre^-Slco2b1^flx/dtr^ BMT	TLF^+^ Cardiac MΦ	Depletion	Peripheral perivascular MΦ	(5)
LysMcre	Gata6^+^ Pericardial MΦ(Gata6^flx/flx^), CCR2^-^ Cardiac MΦ (KLF4^flx/flx^)	Conditional KO	Neutrophils, monocytes	(4, 10, 14, 22, 37)
Lyve1^ncre^-Cx_3_CR1^ccre^	TLF^+^ Cardiac MΦ	Depletion, Conditional KO	Peripheral perivascular MΦ	(38)
CD45^dre^-Gata6^dox/cre^	Gata6^+^ Pericardial MΦ	Depletion, Conditional KO	Other Gata6^+^ cavity MΦ, lymphocytes	(39, 40)

MΦ, macrophage**; **DTR, diphtheria toxin receptor; BMT, bone marrow transfer; TLF^+^, TimD4^+^, Lyve1^+^, and FolR2^+^.

### Exogenous approaches

Non-genetic approaches – utilizing delivery of toxic substances, blocking antibodies and small interfering RNA (siRNA) – have been used to target both CRM and monocyte-derived (recruited) macrophages. They provide the benefit of applying the approach to different transgenic animals including reporter strains. However, their efficiency and specificity depend on their ability to reach the targeted population. Clodronate liposomes have been extensively used to deplete macrophages by inducing apoptosis of the cells upon their phagocytosis of this compound. This has been applied in the context of the heart to deplete macrophages either prior or during cardiac remodeling (27–30). This can provide a global assessment of cardiac macrophages (resident and recruited) but lacks true specificity to target subsets. More recently, antibody or small molecule targeting of the macrophage colony stimulating factor (M-CSF) receptor (anti-CD115) has been shown to effectively deplete CRM following repetitive administration (31, 34). The effectiveness of this approach highlights the dependence of these embryonically derived cells on M-CSF for their maintenance. It should be noted that these approaches could influence monocyte-derived populations, and so the timing is important. Alternatively, monocyte-derived macrophages can be targeted by influencing their ability to mobilize from the bone marrow and/or hinder their recruitment to the heart, both of which rely on CCR2-CCL2 signaling axis. Blockade of CCR2 function through use of an antagonist, blocking antibody or siRNA targeting have all been employed for this purpose (32, 33, 36). In an alternative approach, siRNA targeting of endothelial adhesion molecules has been adopted to achieve the same outcome (41). It is unclear how much these approaches influence CCR2^+^ CRM, particularly under homeostatic conditions, and other circulating immune populations that also express CCR2 as their recruitment may also be affected by these approaches.

### Transgenic approaches

Genetic approaches provide a greater variety of experimental options and can increase the specificity to the CRM and pericardial macrophages being targeted. Global knockout approaches targeting CSF1-CSF1R and CCR2 signalling mimic exogenous methods to target embryonically-derived and monocyte-derived macrophages; however, these methods have been associated with chronic systemic defects (29, 31, 35, 42–45). For example, targeting either the CSF1 gene or CSF1R gene results in depletion of many tissue-resident macrophages and circulating monocytes, but is also associated with growth, development and fertility abnormalities (37). Recent targeted deletion of a CSF1R enhancer region has been able to reverse these defects while still reducing tissue-resident populations including MHCII^hi^ CRM and Gata6^+^ peritoneal macrophages (46). Diphtheria toxin (DT) depleter mice, where the diphtheria toxin receptor (DTR) gene is inserted after a promoter of interest, provides more temporal regulation of depletion. This depletion is under the control of diphtheria toxin treatment, which engages with the diphtheria toxin receptor expressed by the cells of interest to induce their cell death. CD169-DTR, CCR2-DTR, and CD11b-DTR mice systems following diphtheria toxin delivery provide the ability to deplete CCR2^-^ CRM, CCR2^+^ CRM, and all cardiac macrophages, respectively (4, 35, 38, 43).

Cre-Lox systems (constitutive or tamoxifen inducible) are employed for a multitude of applications including DTR mediated depletion, lineage tracing, and conditional gene knockouts. In this system, a mouse strain with a cre-recombinase expressed under the control of a specific promoter is crossed with a mouse that contains cre-recombinase sensitive loxp sites that flank either a stop sequence that sits upstream of an inserted gene segment (e.g. DTR, fluorescent protein) or an endogenous gene of interest. Removal of the loxp flanked segment is achieved through recombination in cells that actively express cre-recombinase. The use of a mutant form of the human estrogen receptor with the cre-recombinase (creER) can further regulate the cre-recombinase activity of the enzyme by holding it in the cytosolic space until tamoxifen engages with the mutated human estrogen receptor, allowing for relocation of the cre-recombinase to the nucleus. This provides additional temporal regulation and has been leveraged to target more long-term CRM. For example, a Cx_3_CR1-creER system along with early in life tamoxifen delivery (3-5 weeks old) and a long wash-out period has been employed to specifically tag long-term CCR2^-^ (TLF^+^, MHCII^hi^) CRM (2, 3, 18). When this strategy is combined with a cre-recombinase sensitive DTR strain, delivery of DT at 8 weeks of age results in efficient depletion of these populations (2). To more specifically target TLF^+^ CRM, Chakarov et al. crossed a Lyve1-cre strain with a solute carrier organic anion transporter family member 2B1(Slco2b1) driven DTR with an upstream loxp-flanked stop sequence. Following the transfer of Lyve1cre-Slco2b1^flox/DTR^ bone marrow into wildtype recipients, DT treatment leads to TLF^+^ CRM depletion upon DT administration while conserving Lyve1 expressing endothelium (5). In addition to these DTR depletion approaches, these Cre-Lox systems have been leveraged to also target expression of specific effector molecules of these macrophage populations. Cx_3_CR1creER has been extensively used in this regard under both homeostatic and pathological conditions (18, 35, 42, 43). Lysozyme M driven cre-recombinase is an alternative approach that has been used to target both cardiac and pericardial macrophages (11, 29). It is important to note that although these promoters narrow the type of cells they target, they are not entirely specific. For example, Cx_3_CR1 is expressed by circulating monocytes and lymphocytes (39, 40), and LysM is expressed by monocytes and neutrophils (47). However, targeting effector molecules specifically expressed by a macrophage subset increases the specificity. For example, a common model to evaluate Gata6 cavity macrophage function is to use a LysMcre-Gata6^fl/fl^ system (11, 15, 48). Since Gata6 is required for maintenance and proliferation, its absence in myeloid cells leads to a relatively specific depletion of Gata6 macrophages including in the pericardial space (11–13, 15, 49).

Despite the improvement in these systems, they are often not selective enough to target the specific subsets. To this end, creation of split or dual recombinase systems are starting to be developed to enhance targeting of specific subsets. Kim et al. developed a binary split cre-recombinase system where the N-terminal portion of cre-recombinase (ncre) is driven by the Lyve1 promoter and the C-terminal portion by the Cx_3_CR1 promoter in order to target perivascular macrophages (Lyve1^+^Cx3CR1^+^) in the central nervous system (50). This construct also resulted in a small proportion of CRM being tagged (50). An alternative strategy used by Jin et al. has employed a dual recombinase system that combines a CD45-driven dre recombinase with a Gata6 driven cre recombinase containing a dre recombinase flanked stop sequence upstream of the cre to target Gata6 cavity macrophage both with reporter and DTR systems (51, 52). For all of these transgenic models, it is important to understand how the genotype influences efficiency of the construct. For example, the labeling of Lyve1^+^ brain perivascular macrophages using the split cre recombinase system was highly increased from 20% to 60% when moving from heterozygous to homozygous carriers for the ncre recombinase fragment (50). Equally as important is how the transgene is inserted – whether directly in the coding sequence or at the end of the coding sequence *via* a cleavable linker protein (e.g. 2A linker). If it is the former, such as the dre recombinase insertion of the CD45 gene in the dual recombinase system (51), careful breeding considerations are needed to avoid a gene knockout that could influence interpretation of macrophage subset function. Further development of these systems with careful selection of appropriate promoters will provide an enhanced toolkit that will allow more targeted assessment of the identified subsets evaluated by single cell sequencing approaches.

## Developmental and homeostatic functions

The development of these outlined tools has provided the opportunity to evaluate the function of these various cardiac and pericardial macrophages. First, during development, Lavine et al. demonstrated a critical role of CRM in coronary artery development. Using a lineage tracing system, they showed that seeded Lyve1^+^ CRM associate with the nascent vessels in the heart and regulate coronary vascular development through insulin growth factor-1 (IGF-1) signaling (44). Cahill et al. have since extended this vascular development function to further show that embryonic CRM also control a subepicardial lymphatic network in the developing heart (53). This is proposed to depend on hyaluronan production by CRM (53). As the mouse reaches adulthood, embryonically-derived Lyve1^+^ (TLF^+^) CRM continue to occupy this perivascular niche and likely contribute to ongoing vascular maintenance (5). In addition to their vascular interactions, CRM directly network with cardiomyocytes to regulate their function and metabolism. Hulsmans et al. identified a high concentration of Cx_3_CR1^+^ CRM in atrial ventricular node of the heart that controls electrical conduction between the atria (top) and ventricles (bottom) of the heart ensuring coordinated contractions of these compartments (35). These CRM directly communicate with their neighbouring cardiomyocytes through the formation of connexin 43 formed connexon channels, which are typically found between cardiomyocytes to facilitate action potential propagation. Global macrophage depletion with the CD11b-DTR or Cx_3_CR1 specific deletion of connexin 43 results in dysregulated electrical activity including blockage of the electrical transmission in the AV node. Cardiac macrophage-derived amphiregulin, which facilitates organization of CX43 connexon channels between cardiomyocytes, could potentially contribute in an autocrine capacity to form CRM-cardiomyocyte connexon channels (54). In fact, deletion of amphiregulin contributes to increased arrythmias including AV block under stress conditions (54). As such, CRM, either through direct or paracrine action, fine tune the electrical modulation of cardiomyocytes within their local environment. While the three main CRM subsets are found in the AV node (35), whether these proposed functions are reserved to a single population or apply to all CRM still remains unclear. As noted above, MHCII^hi^ CRM localize around nerve fibers, supporting a potential role in this regard (5). In addition to electrical conduction, CRM have recently been linked to metabolic regulation of cardiomyocytes (38). Analysis of close interactions between CRM and cardiomyocytes revealed the ability of CRM to sample cardiomyocyte content. Using a mixture of reporter systems, Avila et al. showed the secreted cardiomyocytes structures, termed exophers, represented mitochondrial remnants (38). Exopher production in the heart is dependent on autophagic mechanisms and CCR2^-^ CRM utilize the cell surface receptor MERTK for uptake of these released structures. Depletion of CCR2^-^ CRM using the CD169-DTR system or global deletion of the macrophage restricted MERTK results in increased free mitochondria and impairments in cardiac function (38).

While these studies have revealed the role for resident macrophages in the heart, homeostatic functions in their pericardial counterparts are less clear. One of the key physiological roles of the pericardium is to lubricate the heart. The proteoglycan lubricin, that is abundant in the pericardial space, has been associated with this function (55). In fact, patients with mutations in the lubricin gene develop Camptodactyly-Arthropathy-Coxa vara-Pericarditis (CACP) syndrome, characterized by arthritic issues and an inflammation of the pericardium (pericarditis) (56). Mesothelial cells that line the pericardial space can produce this proteoglycan (55). Recent sequencing of Gata6^+^ pericardial macrophages has revealed lubricin as one of the top expressed genes by this macrophage population (11). Further investigation is required to explore whether production of lubricin represents one of the homeostatic functions of these resident pericardial macrophages.

## Macrophage heterogeneity during cardiac remodeling

Beyond their established roles in maintaining normal heart function, both resident and monocyte-derived macrophages play integral roles in the management and progression of pathological conditions that influence heart function and can lead to maladaptive remodeling, contributing to heart failure. These pathological conditions can be broadly characterized into remodeling in response to cardiomyocyte death or remodeling in the absence of cardiomyocyte death due to peripheral or intrinsic factors. We are now starting to understand that macrophage heterogeneity and recruitment of monocyte-derived macrophages can differ in these different settings and can contribute to different aspects of disease progression.

### Myocardial infarction associated macrophage heterogeneity

The most common clinical form of sterile injury in the heart is a myocardial infarction which typically results from the occlusion of coronary vessels that blocks oxygen and nutrients from reaching downstream heart muscle. This sterile injury stimuli triggers cardiomyocyte death leading to an acute inflammatory response and an extensive remodeling process for which is heavily directed by both CRM and recruited macrophages, as well as pericardial macrophages. Our understanding of the role of macrophages in this context has stemmed from the ligation of the coronary artery in the mouse heart and can be done in a permanent occlusion or an ischemia/reperfusion injury, both of which mimic certain aspects of the human condition. The resulting ischemic injury contributes to early loss of resident CRM in the infarcted zone and leads to replacement by monocyte-derived macrophages mobilized from both the bone marrow and spleen (2, 4, 36, 57, 58). Similar replacement of CRM have also been noted in other models of genetically induced cardiomyocyte death and infectious or autoimmune myocarditis (10, 32, 59). Despite the heavy loss of resident CMs in regions of cardiomyocyte death, these populations play integral roles in shaping the inflammatory and remodeling responses. Deleting CCR2^-^ CRM (TLF^+^, MHCII^hi^ populations) prior to MI using the Cx_3_CR1creER-DTR system, results in more severe fibrosis (2). This may be the result of promoting the pro-inflammatory environment. Using the CD169-DTR model, Bajpai et al. targeted the same CCR2^-^ CRM, resulting in a shift to more pro-inflammatory macrophage profiles in the heart post-MI (4). In a different injury model, specific depletion of perivascular TLF^+^ CRM contributed to increased recruitment of neutrophils and inflammatory cells (5). These beneficial remodeling and immunomodulatory roles for CCR2^-^ CRM at least partly rely on their early efferocytosis of apoptotic cells. This recycling function, mediated by surface receptors such as MERTK and CD36, helps shape the local immune response by promoting effector molecule production. MERTK- and CD36-mediated efferocytosis contributes to increasing CCR2^-^ CRM production of reparative mediators (e.g. IL-10, TGF-B, VEGF-A, VEGF-C) and prevents prolonging the local inflammatory environment (9, 60, 61). TLF^+^ CRM are proposed to have a higher contribution to these mechanisms (9, 61). CRM-dependent MERTK actions are also linked the macrophage protection against ventricular arrythmias, an important complication associated with extensive ventricular tissue damage following MI. Grune et al. recently showed that this is related to maintaining cardiomyocyte mitochondrial function, reminiscent of their homeostatic role (34). Given the overlapping mechanisms involved, it remains unclear what proportion of CCR2^-^ CRM protective effects post-MI relates to their homeostatic function versus their acute functions. Interestingly, the extracellular portion of MERTK can also be cleaved through proteolytic activity shortly following MI, thus impacting these protective effects (9).

While CCR2^-^ CRM diminish at the infarct, recruited monocyte-derived macrophages fill that void and are often described as detrimental to the healing process in the heart following MI. This stems largely from studies showing that depletion of CCR2^+^ macrophages or blocking the recruitment of monocytes through CCR2 targeting contributes to overall improved functional benefit and reduced fibrosis (4, 41, 45, 62). Upon arrival to the infarct, the recruited CCR2^+^ macrophages continue to sustain the phagocytic role started by CCR2^-^ CRM (9), however, contributing to a more inflammatory mediator profile. For example, King et al. demonstrated that recruited macrophages adopt an interferon signaling signature through a STING and cGAS dependent mechanism that senses intracellular double stranded DNA (dsDNA) (63). It is unclear whether the resident ISG CRM also participate in this signaling. Depletion with CCR2^+^ CRM (CCR2-DTR), which include this interferon related gene signature, contributes to shifting the macrophage repertoire to be less inflammatory (4). Blunting of monocyte recruitment in CCR2 deficient animals also contributes to less MERTK cleavage following MI, resulting in enhanced efferocytosis (9). Over time these monocyte-derived macrophages are thought to transition from inflammatory-like cells, that serve as sources of proteases and inflammatory cytokines, to a more repair-like phenotype that promotes angiogenesis and ECM remodeling through the release of multiple factors (64, 65). The orphan receptor NR4A1 has been identified as the important transcriptional mediator of this recruited macrophage polarization (66). This transition is likely to be regulated by the integration of external cues within the local environment. Interactions with other immune populations in the infarct have been shown to heavily influence this polarization process. Neutrophils, which are often associated with inducing further damage of infarcted heart can also serve as important sources of mediators such as annexin A1 (ANX1) and neutrophil gelatinase-associated lipocalin (NGAL) that promote inflammatory resolution. ANX1 stimulates VEGF-A production by local macrophages and NGAL promotes an anti-inflammatory macrophage phenotype and upregulation of MERTK to facilitate efferocytosis (67, 68). Although recruited at much lower levels than neutrophils in the infarcted heart, other granulocyte populations such eosinophils and basophils serve as important local sources of IL-4 and IL-13 that promote cardiac macrophage polarization to a more reparative phenotype (69, 70). These repair-like functions are beneficial early in the remodeling phase, however, ongoing promotion of ECM remodeling can lead to detrimental fibrosis. This is not just limited to the infarcted zone but extends to the remote viable region of the heart where ongoing monocyte recruitment contributes to interstitial fibrosis in these areas (41). This often-accepted view that recruited CCR2^+^ cardiac macrophages are inherently detrimental to post-MI cardiac remodeling is likely an oversimplification. Similar to CRM described above, monocyte-derived cells also provide protection in ventricular arrythmias (34). This function is dependent on CD36 and likely related to mediated cardiomyocyte mitochondrial health, again reminiscent of homeostatic macrophage function in exopher recycling. Beyond these natural protective functions, Vagnozzi et al. have also shown that stimulation of monocyte recruitment to the infarcted heart can promote improved remodeling and propose this a key mechanism for the benefits associated with stem cell therapy in the heart (71). Whether these protective functions reflect monocyte specific or monocyte-derived macrophage mechanisms remains unclear. Dick et al. have also identified the appearance of multiple recruited macrophage populations in the heart, suggesting potential multiple functional phenotypes present in this tissue environment. Understanding what these populations are doing and their location of action will important moving forward.

The blood is not the only source of recruited macrophages in the heart. In fact, Gata6^+^ pericardial macrophages also relocate to the epicardial surface of the heart rapidly following MI and persist for up to 7 days after the injury (11). This was further validated by the newly developed dual recombinase system that allows for true lineage tracing capabilities. Using this approach, Gata6^+^ pericardial macrophages remained in the thickened epicardial surface in the infarcted and border zones at day 7 post-MI (52). This migration to the heart in the mouse coincides with a decrease in their presence within the pericardial fluid (11). Interestingly, a decrease in comparable CD163^hi^ pericardial macrophage numbers in patients is also noted within the first few days (<4 days) following MI, supporting this relocation to the heart as a potential clinically relevant mechanism (22). The function of these cells remains debated. Chronic depletion of these Gata6 pericardial macrophages using the LysMcre-Gata6^flx/flx^ mouse results in the mice developing stiffer hearts and is associated with enhanced fibrosis, particularly in the viable regions of the hearts (11). However, acute depletion of these cells using the dual recombinase DTR system results in only a modest impact on the remodeling heart (52). This perhaps suggest that this fibrosis regulation could be more related to their homeostatic function as opposed to their active relocation to the heart. More detailed experimental work is needed to untangle their local functions in the heart.

In contrast to the adult condition described above, the neonatal mouse heart has the ability to regenerate, and macrophages are central to this capacity. Using clodronate liposomes in a neonatal MI model, Aurora et al. found that depletion of macrophages impeded cardiac regeneration and angiogenesis compared to controls, with substantial differences observed by day 7 post-MI (28). It has been posited that macrophage heterogeneity – specifically, the distinction between neonatal and adult cardiac macrophage populations – influences regeneration (27, 28). The neonatal heart contains only one macrophage population, CCR2^-^ CRM (MHCII^low^CCR2^−^), while the adult heart, contains both CCR2^-^ and CCR2^+^ CRM (27). After cardiomyocyte injury in a genetic ablation model, Lavine et al. identified neonatal hearts had expanded MHCII^low^CCR2^−^ CRM, while adult hearts had selective expansion of MHCII^high^CCR2^+^ recruited macrophages (27). Further assessments of these subsets show that neonatal CCR2^-^ but not adult CCR2^+^ macrophages provided reparative benefit such as stimulated endothelial cell tube formation and cardiomyocyte proliferation (27). Further, transplantation of cardiac macrophages from apical resection-injured neonatal into MI-injured adult mice effectively stimulates both cardiac repair and cardiomyocyte proliferation (72). Beyond mammals, zebrafish experimental models further corroborate a unique role for heterogenous cardiac macrophages in regeneration (73, 74). Similar to what has been identified in neonatal mice, unique subsets of macrophages appear to be implicated in zebrafish cardiac regeneration. While the role of individual macrophage subsets is largely undefined (e.g., *mpeg1* ^+^ *csf1ra* ^+^ compared to *mpeg1* ^+^ *csf1ra* ^-^), cardiac macrophage depletion and ablation in larval and adult zebrafish, respectively, has been found to impede scar resolution, cardiomyocyte proliferation and overall cardiac regeneration (73, 74). In larval zebrafish, macrophages appear to activate the epicardium which, in turn, upregulate *Vegfaa* expression to facilitate cardiomyocyte proliferation, associated with endocardial notch signalling (73). Adult zebrafish also appear to rely on macrophages in a *csf1ra*-dependent manner. Specifically, cryo-injured zebrafish with a point mutation in the c*sf1ra* gene have decreased *tnfa+* but increased *spp1+* cardiac macrophages which appear to coincide with scar resolution and regeneration (74). Further experimental work is required to delineate the specific mechanisms underlying the role of these macrophages; yet, collectively, work in both zebrafish and neonate mice provide further insight into our emerging understanding of macrophages in cardiac regeneration.

### Non-injury remodeling and cardiac macrophage heterogeneity

Beyond ischemic injury, the heart can also undergo dramatic cardiac remodeling due to intrinsic and external peripheral factors that can eventually contribute to non-ischemic cardiomyopathy. A common clinical driver of this type of pathology is high blood pressure that forces the heart to hypertrophy due to the increased workload and is chronically associated with the development of fibrosis. The transaortic constriction (TAC) model and angiotensin II (AngII) infusion models represent common approaches to study this non-injurious cardiac remodeling in mice. Similar to the myocardial infarction, the TAC model elicits an early increase in cardiac macrophages within the first week (29, 31, 33). This increase has been shown to rely on local proliferation of CCR2^-^ CRM and on CCR2 dependent mechanisms depending on the study (29, 31, 33). Interestingly, Gata6^+^ pericardial macrophages minimally accumulate on the surface of heart and do not infiltrate the myocardium in this model (52). Depletion of CCR2^-^ CRM(TLF^+^, MHCII^hi^) using the anti-CD115 antibody results in a sustained deficiency of total macrophages for at least a week following TAC induction with recovery occurring by 2 weeks. This early depletion contributes to long-term increased fibrosis, decreased angiogenesis, and cardiac dysfunction (31). A similar phenotype is observed when cardiac macrophages are depleted with clodronate liposomes and their proliferation is dampened using a KLF4 conditional knockout model (LysMcre-KLF4^flx/flx^) (29). Conversely recruited macrophages counteract the effects of CCR2^-^ CRM. Blocking recruitment of macrophages to the heart using either a CCR2 antagonist or blocking antibody (MC31) within the first week results in the opposite phenotype characterized by lower fibrosis, reduced hypertrophy, preserved cardiac function and less T cell expansion (33).

Macrophage heterogeneity and kinetics follow a different progression in the AngII-induced cardiac hypertrophy model. Using the Cx3CR1creER-tdtomato lineage tracing system, Zyman et al. show that early expansion in tdTomato^-^ cardiac macrophages (mostly recruited CCR2^+^) within the first few days is followed by a progressive expansion of the tdTomato^+^ CCR2^-^ CRM (TLF^+^, MHCII^hi^) (18). Similar to the TAC model, depletion of CCR2^-^ CRM *via* DTR targeting of Cx_3_CR1 pulse chase model results in worse fibrosis and decrease in cardiac function. Interestingly in contrast to the TAC model, CCR2^-^ CRM are direct regulators of the hypertrophic response in the AngII model and IGF-1 produced by these macrophages is a key mediator for this function (18, 31). IGF1 is expressed by CCR2^-^ CRM under steady state conditions and increased under hypertensive stress (18). A complimentary study found that IGF-1 expression in CRM is in part regulated by mechanical stretch. Using a genetic dilated cardiomyopathy model where CCR2^-^ CRM are needed for beneficial adaptation and angiogenesis, CCR2^-^ CRM but not CCR2^+^ cardiac macrophages were shown to directly interact with neighbouring cardiomyocytes and thus form an integrated network within the heart wall (75). An increase in mechanical stretch stimulates activation of TRPV4 channels in these CRM that lead to downstream production of IGF1 (75). Further, inhibition of the TRPV4 blunted the adaptive angiogenic response and remodeling in this model (75). The mechanisms are relevant to the human context, as Zaman et al. also highlighted the presence of IGF-1 expressing cardiac macrophages in both the neonatal and hypertrophic failing heart in patients (18).

In addition to blood pressure, cardiac macrophages respond to multiple systemic changes that can shape the local cardiac environment. Although bacterial uptake is often associated with other tissue-resident macrophages in the lung (Alveolar macrophages) and liver (Kupffer cells), CRM in the heart can phagocytose circulating bacteria (42). Under sepsis-like conditions, CRM (CCR2^-^ and CCR2^+^) undergo local expansion and protect against cardiomyocytes death. These CRM upregulate IL-10 production following bacterial encounter to preserve cardiomyocytes (42). While protective in this circumstance, enhanced IL-10 production by CRM can also be detrimental. In a model of cardiac diastolic dysfunction, CRM derived IL-10 stimulates a fibrotic response by serving as an autocrine factor to promote local osteopontin and TGF-β production leading to fibroblasts activation and ECM production (43). Both CRM (mostly CCR2^-^) and recruited CCR2^+^ cardiac macrophages are present in these models and they both appear to contribute to local IL-10 production, although their relative contributions are not clear. Interestingly, Cx_3_CR1 CreER targeted deletion of IL-10 in both studies results in a reduction in the proportion of MHCII^+^ cardiac macrophages (CCR2 status unspecified), perhaps suggesting an additional role for IL-10 in cardiac macrophage subset maintenance or differentiation. Collectively, these studies again highlight the central role for cardiac macrophages controlling the local cardiac homeostasis and remodeling.

## Future directions

Our understanding of macrophage heterogeneity in and around the heart has grown tremendously under both homeostatic and pathophysiological settings. These advancements have largely been aided by the adoption of transgenic mouse approaches and the increased application of single cell technology in both the mouse and human systems. This abundance of new information can be utilized to further refine our understanding of macrophage biology and function in the cardiac context. For example, the integration of new dual or split recombinase genetic approaches that are starting to be developed can be utilized to target the multiple subsets more specifically and to build upon the functions that have more broadly been associated with either CCR2^-^ and CCR2^+^ cardiac macrophages. A key aspect that has largely been overlooked is the spatial dimension in all of this and how interactions with local cells shape the phenotype and function of these populations. Advances in both spatial scRNAseq, high dimensional histo-cytometry, as well as imaging approaches (e.g. intravital, cleared tissue imaging) should help to better define these local milieus. In addition to the cardiac environment proper, a deeper exploration of communication at the epicardial interface between the heart and pericardial space is warranted. As noted above, the epicardium serves as an important seeding site for the cells of the developing heart including macrophages and is implicated in beneficial remodeling response in the heart. How the neighboring immune cells including macrophage in the pericardial cavity interact with the heart at the epicardial surface remains largely unexplored. Integrating this spatial context will also be important moving forward. It should equally be acknowledged that the mouse systems that have been used to define many of these paradigms are controlled models. Relative to patients suffering from cardiac diseases, the mice used for most studies are young relative to the human context and are typically not exposed to the same stresses. In addition, to limit variability many studies including our own have uniquely focused on an individual sex, however, there is increased awareness for inclusion sex difference inclusion to increase the translational application of these animal models. In the heart context, the acute inflammatory and remodeling is known to differ between men and women, and females typically suffer MIs later in life with a higher propensity developing heart failure as a result. Given our basic understanding of the central role of macrophages in cardiac physiology and remodeling, it would be intriguing to explore how factors such as age and sex impact cardiac and pericardial macrophage subsets and functions.

## Author contributions

CI wrote, edited and prepared the figure and table. JD wrote and edited the article. All authors contributed to the article and approved the submitted version.
