# Supplementary material for: The role of macrophage subsets in and around the heart in modulating cardiac homeostasis and pathophysiology
Source: Front Immunol. 2023 Feb 28;14:1111819. doi: 10.3389/fimmu.2023.1111819 (PMC10011174; doi:10.3389/fimmu.2023.1111819)
Supplement: Supplementary file 1 [file DataSheet_1.pdf]

Supplementary Table 1- Cardiac macrophage focused scRNAseq studies in mouse and human

NCBI GEO ID or HCA DCP ID	Disease	Sample Donor(s)	Timeframe	References
Mouse Datasets				
Contact Author	IR	Control male and female mice	Samples obtained 4 days post-IR injury	Bajpai et al 2018
		CCR2-DTR, CD169-DTR male and female mice	DT administered 4 days before IR injury; Samples obtained 4 days post-IR injury	
GSE119355	Naïve	Control male and female adult mice	Samples obtained at ~20 weeks of age	Dick et al 2019
	MI	Cx3cr1CreER/+:R26Td/+ male and female adult mice	Samples obtained at ~20 weeks of age at day 11 post-MI	
GSE188647	Naïve	Cx3cr1CreER/+:R26Td/+ male and female adult mice	Samples obtained at ~20 weeks of age	Dick et al 2022
	Parabiosis	CD45.1 & CD45.2 male and female adult mice	Mice paired at 7 weeks; Samples obtained at 5- or 25-week timepoints	
GSE86310	Naïve	C57BL/6 male and female adult mice	Samples obtained between 8-40 weeks of age	Hulsmans et al 2017
GSE106473	MI	Control male adult mice	Samples obtained at day 4 post-MI	King et al 2017
		Irf3−/− male adult mice	Samples obtained at day 4 post-MI	
GSE179276	Sham	C57/BL6 male adult mice	Samples obtained after sham surgery	Revelo et al 2021
	TAC	C57/BL6 male adult mice	Samples obtained at day 7 post-TAC	
GSE179343	Sham	Cx3cr1CreER/+:R26Td/+ male and female mice	Samples obtained after sham surgery	Zaman et al 2021
	AngII Infusion	Cx3cr1CreER/+:R26Td/+ male and female mice	Samples obtained at day 4, or day 28 of AngII infusion	
Human Datasets				
GSE188647	Ventricular Septal Defect	Male	Samples obtained from a 12 year-old patient	Dick et al 2022
GSE183852	Healthy and Heart Failure Patients	Male and Females	Samples obtained from healthy donors or non-ischemic heart failure patients aged 8-75	Koenig et al 2022
ERP123138	Post-Mortem (circulatory or brain death)	Males and Females	Samples obtained from transplant donors aged 40-75	Litviňuková et al 2020
GSE179343	Tetralogy of Fallot	Male	Samples obtained from a 5 month-old patient undergoing corrective surgery	Zaman et al 2021
	Hypertrophic cardiomyopathy	Male	Samples obtained from a 46 year-old patient undergoing heart transplant	

AngII: angiotensin II; CV: cardiovascular; DT; diphtheria toxin; DTR: diphtheria toxin receptor; IR: ischemia/reperfusion; LVAD: left ventricular assist device; MI: myocardial infarction; TAC: transverse aortic constriction
